# ZEB1 serves as an oncogene in acute myeloid leukaemia via regulating the PTEN/PI3K/AKT signalling pathway by combining with P53

**DOI:** 10.1111/jcmm.16539

**Published:** 2021-05-07

**Authors:** Lanlan Li, Yubin Feng, Shuang Hu, Yan Du, Xiaoling Xu, Meiju Zhang, Xiaoqing Peng, Feihu Chen

**Affiliations:** ^1^ The Key Laboratory of Major Autoimmune Diseases of Anhui Province Anhui Institute of Innovative Drugs School of Pharmacy Anhui Medical University Hefei China; ^2^ The Key Laboratory of Anti‐inflammatory and Immune Medicines Ministry of Education Hefei China

**Keywords:** acute myeloid leukaemia, AKT pathway, differentiation, P53, PI3K, proliferation, PTEN, ZEB1

## Abstract

Acute myeloid leukaemia is a complex, highly aggressive hematopoietic disorder. Currently, in spite of great advances in radiotherapy and chemotherapy, the prognosis for AML patients with initial treatment failure is still poor. Therefore, the need for novel and efficient therapies to improve AML treatment outcome has become desperately urgent. In this study, we identified the expression of ZEB1 (a transcription factor) and focused on its possible role and mechanisms in the progression of AML. According to the data provided by the Gene Expression Profiling Interactive Analysis (GEPIA), high expression of ZEB1 closely correlates with poor prognosis in AML patients. Additionally, the overexpression of ZEB1 was observed in both AML patients and cell lines. Further functional experiments showed that ZEB1 depletion can induce AML differentiation and inhibit AML proliferation in vitro and in vivo. Moreover, ZEB1 expression was negatively correlated with tumour suppressor P53 expression and ZEB1 can directly bind to P53. Our results also revealed that ZEB1 can regulate PTEN/PI3K/AKT signalling pathway. The inhibitory effect of ZEB1 silencing on PTEN/PI3K/AKT signalling pathway could be significantly reversed by P53 small interfering RNA treatment. Overall, the present data indicated that ZEB1 may be a promising therapeutic target for AML treatment or a potential biomarker for diagnosis and prognosis.

## 1 INTRODUCTION

Uncontrolled proliferation and maturation of hematopoietic stem cells are the characteristics of acute myeloid leukaemia (AML), which is the most common and severe form of acute leukaemia in adults.^1^, ^2^ Although great progress has been made in the diagnosis and treatment of AML, the overall 5‐year survival rate of patients is still very low due to high recurrence rate and chemical resistance, which is considered as the main obstacle of AML treatment.^3^, ^4^, ^5^, ^6^, ^7^ Consequently, identifying novel therapeutic strategies to prevent or treat of AML is considerably needed.

Epithelial‐mesenchymal transition (EMT) is considered to be a critical process in normal embryonic development, which has also been revealed to be involved in the tumorigenesis, metastasis and drug resistance.^8^ Zinc finger E‐box‐binding homeobox 1 (ZEB1), a member of the zinc finger transcription factor family, regulates the expression of epithelial/mesenchymal markers (such as E‐cadherin, N‐cadherin and vimentin) to modulate the EMT progression.^9^ Up‐regulation of ZEB1 has been found in a variety of tumours and positively correlated with the degree of malignancy and poor prognosis.^10^ Previous studies have shown that ZEB1 acts with other transcriptional regulators and regulates tumour metastasis via EMT in lung cancer,^11^ hepatoma,^12^ breast cancer^12^, ^13^ and colorectal cancer.^14^, ^15^ In addition, ZEB1 is implicated in the regulation of proliferation and differentiation of various cells, such as neuronal progenitors,^16^ vascular smooth muscle cells^17^ and osteoblasts.^18^ Wafaa Ghoneim Shousha al^19^ reports that ZEB1 has potential as diagnostic and prognostic marker for AML. However, the specific molecular mechanism has not been clarified.

P53 serves as a well‐known tumour suppressor that is implicated in tumorigenesis and tumour progression. It is reported that the expression and function of P53 can be directly or indirectly affected by several zinc finger transcription factors, including ZEB1, Snail and Slug. Recently, the findings of Fu et al showed that ZEB1 deletion results in the stabilization and activation of p53 protein and thereby inhibiting mammary epithelial tumours growth and progression.^20^

The PTEN/PI3K/AKT signalling pathway plays a vital role in the initiation and progression of multiple tumours by modulating cell proliferation, differentiation, invasion and metastasis.^21^ In a recent study, miR‐205 suppresses the activation of AKT/mTOR signalling pathway by down‐regulation of ZEB1, reverses EMT and thereby inhibits glioblastoma cells migration and invasion.^22^ In addition, PTP4A1 promotes proliferation and invasion in intrahepatic cholangiocarcinoma via up‐regulating ZEB1, accompanied by the activation of PI3K/AKT pathway.^23^ In addition, P53 can regulate the PTEN/PI3K/AKT signalling pathway.^24^ Based on the above observations, the present study was conducted to explore whether ZEB1 regulated the PTEN/PI3K/AKT signalling pathway by associating with P53 in AML.

## 2 MATERIALS AND METHODS

### 2.1 Cell culture

Normal monocyte cell line SC, acute myeloid leukaemia cell lines NB4, K562 and THP‐1 were all purchased from the Shanghai Institute of Cell Biology, Chinese Academy of Sciences and cultured in our laboratory under standard conditions. The medium was changed every 24 hours, and the subculture cells were changed every 48 hours. Scrambled siRNA, ZEB1 siRNA and P53 siRNA were synthesized by GeneChem (Shanghai, China). GV141‐ZEB1 and empty GV141‐control were also designed by GeneChem. Transfection of cells was conducted using Lipofectamine™ 2000 (Invitrogen, USA) according to the manufacturer's protocol. The medium was exchanged 6 hours after transfection. All experiments were repeated at least three times.

### 2.2 Western blot analysis

Total lysates from cells or tissue samples were extracted using RIPA lysis buffer (Beyotime) containing protease inhibitor cocktail. After being mixed with 5× loading buffer, samples were boiled at 100°C for 10 minutes and then subjected to SDS/PAGE on a 10% gel. Protein signals were visualized using enhanced chemiluminescence (ECL) system (Thermo Fisher Scientific, Rochester, NY). The primary antibodies used were as follows: ZEB1, P53, CDK4, cyclin A2, cyclin D1, p‐Rb, CD11b, CD14, PI3K, p‐PI3K, AKT, p‐AKT and PTEN. All antibodies were from Abcam, Danvers, MA, USA and diluted 1:1000. The primary antibody for β‐actin (Bioss, Beijing, China) was used at a 1:500 dilution.

### 2.3 Differentiation marker analysis

After the respective treatment, cells were harvested and washed twice with pre‐cooling PBS, followed by incubation with 1 µL CD11b‐PE/CD14‐FITC antibody for 30 minutes at 4°C and protected from light. Finally, the samples were analysed by a CytoFLEX flow cytometer (Beckman Coulter).

### 2.4 Cell cycle analysis

After the respective treatment, cells were harvested and washed twice with pre‐cooling PBS and then fixed in 75% cold ethanol at −20℃ overnight. After centrifugation at 1000 × *g* for 10 minutes, the cells were resuspended in PBS containing RNAse A (20 μg /mL) for 30 minutes at 37°C water bath, and then, 400 µl PI was added and incubated on ice for 30 minutes. Stained cells were evaluated by a flow cytometry (Becton Dickinson) to determine cell cycle distribution. Data were analysed using ModFit software (Verity Software House).

### 2.5 Double‐immunofluorescent staining

Double‐immunofluorescent staining was performed to assess colocalization of ZEB1 and P53. Cells were plated in six‐well plates and fixed with 4% paraformaldehyde for 10 minutes, blocked with 10% BSA for 10 minutes and incubated with primary antibodies and secondary antibody, respectively. Afterwards, DAPI was used to stain the nucleus and cells were observed under a laser scanning confocal microscope.

### 2.6 Tumour xenografts

6‐week‐old male NCG nude mice were purchased from the Nanjing Model Animal Institute. The mice were fed in SPF centre of Anhui Medical University (Hefei, China) for 1 week to adapt new environment before the beginning of the experiment. The mice were then randomly classified into two groups (n = 6), 1 × 10^6^ stably transfected NB4 cells were implanted into the armpit of the mice via subcutaneous injection. Finally, the tumour‐bearing mice were sacrificed, and the tumours from each group were photographed and weighed. The present study was approved by the Ethics and Research Committees of Anhui Medical University and performed in accordance with the National Institutes of Health Guide for the Care and Use of Laboratory Animals.

### 2.7 Samples

Bone human bone marrow specimens from newly diagnosed AML patients and healthy volunteers were obtained from the First Affiliated Hospital of Anhui Medical University. 3–5 mL of the bone marrow was collected by bone marrow puncture. Bone marrow mononuclear cells (BMMC) from each sample were then isolated by standard Ficoll‐Hypaque density centrifugation. The present study was approved by the Ethics and Research Committees of the First Affiliated Hospital of Anhui Medical University. Written informed consent was get from all subjects in accordance with the Declaration of Helsinki.

### 2.8 Co‐immunoprecipitation (Co‐IP) assay

The co‐immunoprecipitation assay (Co‐IP) assay was conducted using the Co‐IP Pull‐Down Kit (Ribobio Guangzhou) according to the manufacturer's protocol. NB4 and THP‐1 cells were lysed in prechilled lysis buffer containing phosphatase inhibitors and protease inhibitors. Lysates were incubated with protein A/G beads and anti‐ZEB1 antibodies with gentle shaking overnight at 4°C. Beads were then washed extensively, eluted with the buffer and analysed by an immunoblot analysis as previously described.

### 2.9 Statistical analysis

SPSS software (version 19.0) was used for statistical analysis of data, and all values are represented by mean ± standard deviation (SD). Students' t test was used for comparison among groups, and single ANOVA was used for comparison among multiple groups, *P* < 0.05 was considered statistically significant (*P < 0.05, **P < 0.01).

## 3 RESULTS

### 3.1 ZEB1 was up‐regulated in AML and suggests poor prognosis

**FIGURE 1:**
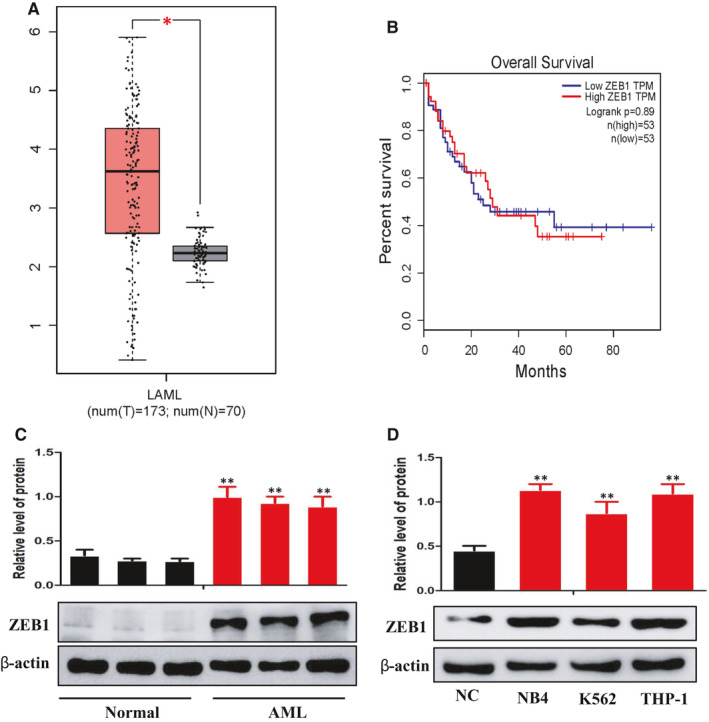
ZEB1 is aberrantly overexpressed in AML and is associated with poor prognosis. A, Box plots for ZEB1 gene expression in AML and normal tissues from GEPIA. B, Kaplan‐Meier survival plot of overall survival of AML patients in GEPIA, categorized according to ZEB1 gene expression (high vs. low, based on mean expression). C, Western blot analysis of ZEB1 protein levels in AML and normal tissues. D, Western blot analysis of ZEB1 expression in leukaemia cell lines and SC cells. β‐Actin served as a loading control. Data are presented as the mean ± SD of three independent experiments. *P < 0.05, **P < 0.01, compared with the normal group

To investigate potential significance of ZEB1 in AML, we first analysed the expression levels of ZEB1 in AML using the available data sets from the GEPIA database (http://gepia.cancer‐pku.cn/detail.php). Relatively high level of ZEB1 was detected in AML patients (left bar) compared with normal cases (right bar) (Figure 1A). Importantly, Kaplan‐Meier analysis demonstrated that AML patients with overexpression of ZEB1 exhibited worse overall survival (Figure 1B). These results indicate a possible link between ZEB1 and AML progression. To further confirm our findings, the Western blot analyses were conducted to evaluated ZEB1 expression in AML patients and the healthy controls. We found AML group have higher ZEB1 levels than the healthy group (Figure 1C). In addition, compared with normal human monocytes, multiple leukaemia cell lines, such as NB4, THP‐1 and K562, exhibited a significantly elevated protein level of ZEB1 (Figure 1D). From this, we could infer that ZEB1 is closely related to AML progression.

### 3.2 Down‐regulation of ZEB1 inhibited proliferation and induced differentiation of AML cells in vitro

**FIGURE 2:**
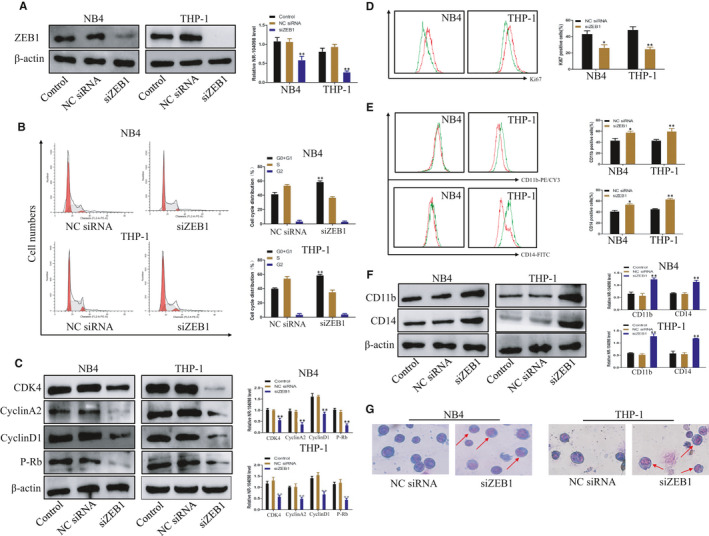
Down‐regulation of ZEB1 inhibited proliferation and induced differentiation of AML cells in vitro. After transfection with siRNA for 72 h. A protein levels of ZEB1 were detected in NB4 and THP‐1 cells without transfection (lane 1) or transfection with negative control (lane 2) or si‐ZEB1 (lane 3). B Knock‐down of ZEB1 induced G0/G1 phase arrest in NB4 and THP‐1 cells according to flow cytometry analysis. C Protein levels of cyclin D1, cyclin A2, P‐Rb and CDK4 were detected in NB4 and THP‐1 cells without transfection (lane 1) or transfection with negative control (lane 2) or si‐ZEB1 (lane 3). D Knock‐down of ZEB1 inhibited ki67 expression in NB4 and THP‐1 cells according to the flow cytometry analysis. E Knock‐down of ZEB1 induced differentiation in NB4 and THP‐1 cells according to the flow cytometry analysis. E Protein levels of CD11b and CD14 were detected in NB4 and THP‐1 cells without transfection (lane 1) or transfection with negative control (lane 2) or si‐ZEB1 (lane 3). F Wright‐Giemsa staining for cell morphology. β‐actin was used as the loading control. Data are presented as the mean ± SD of three independent experiments. *P < 0.05, **P < 0.01, compared with the NC group

Considering high ZEB1 expression in AML, we further explored its role in the proliferation and differentiation of AML cells. First, the siRNA‐ZEB1 or negative control (siRNA‐NC) was transfected into NB4 and THP‐1 cell lines. As shown in Figure 2A, silencing efficiency was confirmed by Western blotting. We next performed a series of experiments to explore the functional significance of ZEB1. Flow cytometric assays showed ZEB1 silencing resulted in increased G0/G1 phase arrest and decreased the population of S phase (Figure 2B). Consistently, the cell cycle markers (CDK4, cyclin A2, cyclin D1, p‐Rb) were detected to be down‐regulated in ZEB1‐silenced group (Figure 2C). Flow cytometry also showed that ZEB1 could inhibit proliferation‐related protein Ki67 (Figure 2D). To further study the effects of ZEB1 on cell differentiation, the flow cytometry (Figure 2E) and Western blot analysis (Figure 2F) were performed, showing that knock‐down of ZEB1 promoted CD11b and CD14 (relatively classic markers of differentiation) expression in NB4 and THP‐1 cells. In addition, similar results were obtained when cell morphology was evaluated using Wright‐Giemsa staining (Figure 2G). In the si‐ZEB1 group, cells were characterized by matured appearances that were the smaller nucleoli, the decreased nuclear/cytoplasm ratio. Taken together, these data above indicated that silencing of ZEB1 suppressed AML proliferation and promoted differentiation.

### 3.3 ZEB1 can interact with P53 and regulate its expression in AML cells

**FIGURE 3:**
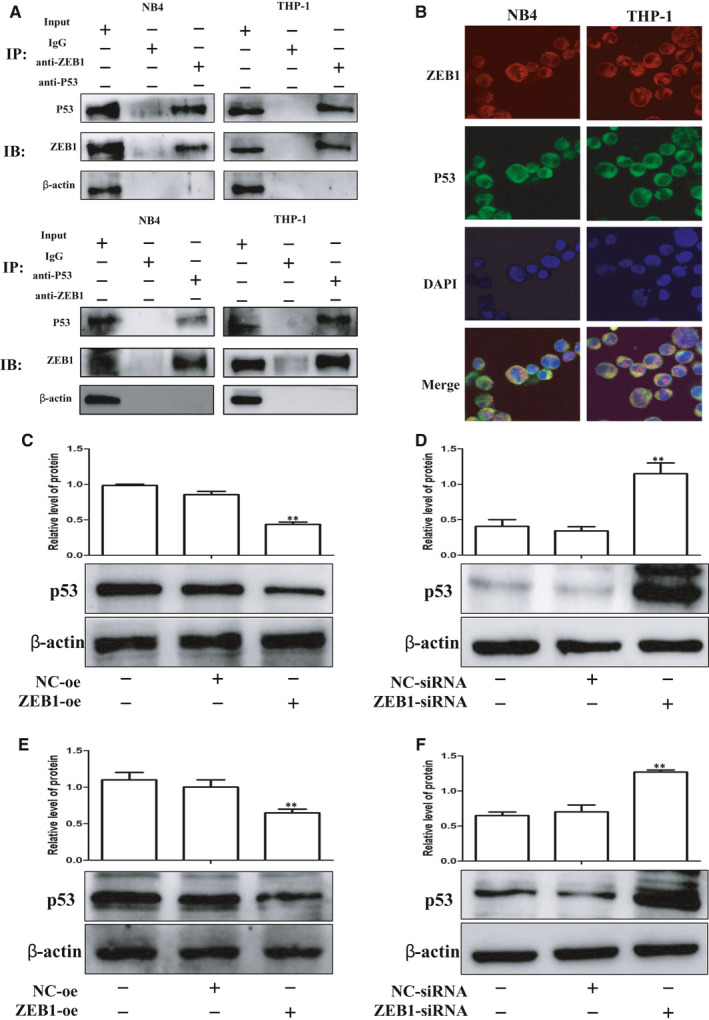
ZEB1 can bind to P53 and regulate its expression in AML cells. A The binding of ZEB1 and P53 in the immunoprecipitation complex was validated by Western blotting. B ZEB1 and P53 colocalized to the cytoplasm. After transfection with plasmid for 72 h. C, E, Western blot analysis of P53 protein levels in NB4 and THP‐1 cells after ZEB1 overexpression. After transfection with siRNA for 72 h. D, F, Western blot analysis of P53 protein levels in NB4 and THP‐1 cells after ZEB1 depletion. β‐actin was used as the loading control. Data are presented as the mean ± SD of three independent experiments. *P < 0.05, **P < 0.01, compared with the NC group

All above data suggested that ZEB1 was essential for the differentiation and proliferation of AML. We next sought to identify the involved molecular mechanisms. In order to verify the interaction between ZEB1 and P53, double‐immunofluorescent staining Co‐IP and were performed. The Co‐IP experiments in AML cells revealed apparent binding of ZEB1 and P53 (Figure 3A). According to the combined fluorescence images, obvious colocalization of ZEB1 and P53 was found in the cytoplasm (Figure 3B). Finally, we examined the correlations between ZEB1 and P53 at protein levels. Western blotting showed that ZEB1 silencing promoted the expression of P53 (Figure 3D, F), whereas ZEB1 overexpression inhibited the expression of P53 (Figure 3C, E). Altogether, ZEB1 could bind to P53 and regulate its expression.

### 3.4 The regulatory function of ZEB1 on PTEN/PI3K/AKT signalling pathway can be reversed by knock‐down of p53

**FIGURE 4:**
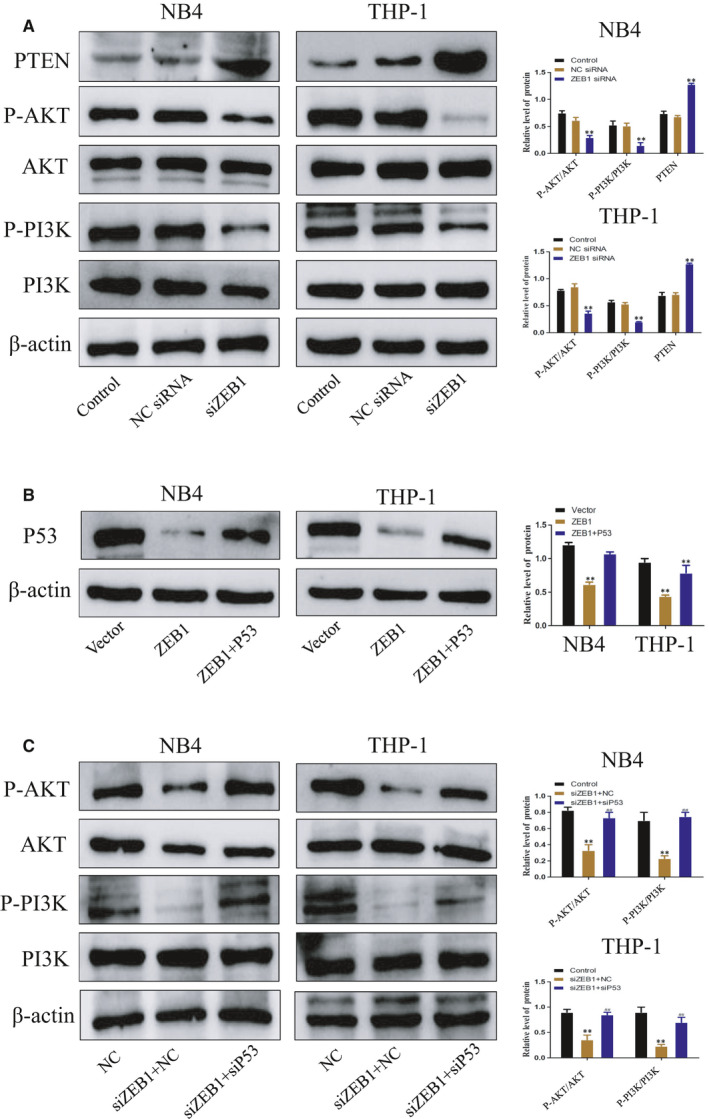
Knock‐down of P53 reverses the inhibitory effect of ZEB1 on the PTEN/PI3K/AKT signalling pathway. After transfection with siRNA for 72 h. A Western blot analysis of PTEN, AKT, p‐AKT, PI3K and p‐PI3K in NB4 and THP‐1 cells without transfection (lane 1) or transfection with negative control (lane 2) or si‐ZEB1 (lane 3). B PTEN, AKT, p‐AKT, PI3K and p‐PI3K levels in NB4 and THP‐1 cells were measured by Western blotting. Data are presented as the mean ± SD of three independent experiments. *P < 0.05, **P < .01, compared with the NC group. ^#^ *P* < 0.05, ^# #^ *P* < 0.01, compared with the siZEB1 + NC siRNA

PTEN/PI3K/AKT is an important intracellular signalling pathway, participating in the regulation of various cellular functions such as cell proliferation, differentiation, apoptosis and glucose transport. In view of the above results, we explored whether ZEB1 exerted its effect via the PTEN/PI3K/AKT pathway. Western blot analysis revealed that the protein levels of PTEN, p‐PI3K and p‐AKT were significantly reversed in ZEB1 silenced cells (Figure 4A). As expected, P53 knock‐down also reversed the inhibition of PTEN/PI3K/AKT signalling by ZEB1 silencing in AML cell lines (Figure 4B). Taken together, ZEB1 might mediate the PTEN/PI3K/AKT signalling by p53 in AML cells.

### 3.5 Altered ZEB1 expression affects tumour growth in vivo

**FIGURE 5:**
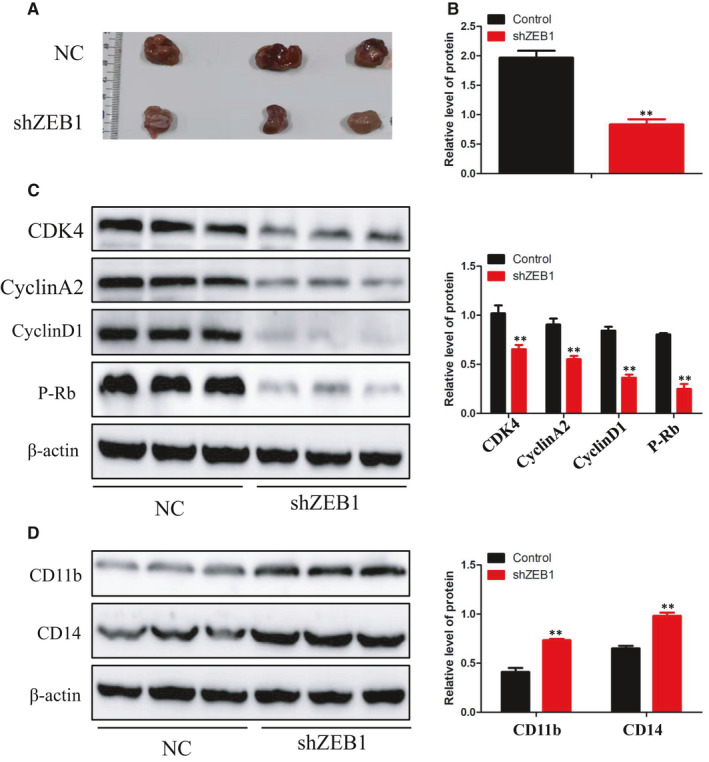
Effects of ZEB1 on tumour growth in vivo. A, B, Tumour images and weights at experimental end‐points in NC and shZEB1 NB4 xenografts (n = 5 for each group). C, Western blot analysis of cyclin D1, cyclin A2, P‐Rb and CDK4 in tumour tissues of NC and shZEB1 groups. D, Western blot analysis of CD11b and CD14 in tumour tissues of NC and shE2F4 groups. β‐actin was used as an internal control. Bar graphs (mean ± SD) and representative images are shown. *P < 0.05, **P < 0.01, compared with the NC group

Based on the data obtained from the vitro experiments, we further studied the effects of ZEB1 on tumour growth in vivo. NB4 cells (1 × 10^6^), stably transfected with ZEB1 shRNA or with control shRNA, were subcutaneously injected into NCG mice (6 mice for each group). Tumour growth was closely monitored for 3 weeks. At the end of week 3, mice were killed and tumours were excised, measured, weighed, photographed and analysed. Photographs of tumours (Figure 5A) and tumour weights (Figure 5B) showed that ZEB1 silencing inhibited tumour cell growth. Compared with the NC tumours, the protein level of CD11b and CD14 greatly increased (Figure 5D) in ZEB1 knock‐down tumours, while the expression of cyclin D1, cyclin A2, p‐Rb and CDK4 was markedly decreased (Figure 5C) in ZEB1 knock‐down tumours. These results indicated that knock‐down of ZEB1 inhibited the development of tumour in vivo.

## 4 DISCUSSION

AML, a genetically and clinically heterogeneous haematological disease, still remains a great therapeutic challenge until now.^25^, ^26^, ^27^ A majority of patients with AML develop tumour relapse after initial treatment and eventually succumb to related complications such as infection, bleeding and organ failure. Thus, there is an urgent need to identify new and effective molecular targets for AML. In this study, we found that the expression of ZEB1 was significantly higher in AML patients and several leukaemia cell lines compared with normal control by analysing the data in public databases and Western blot. The GEPIA database analysis also confirmed that the high expression levels of ZEB1 in patients with AML was associated with markedly worse patient overall survival. These results provided powerful evidence that ZEB1 might have an oncogenic role in AML.

The well‐established features of leukaemia are uncontrolled proliferation and impaired differentiation. The human cell cycle is a complex cell activity which results in cell division and duplication. Cell cycle arrest is one of the indicators of proliferation inhibition and occurs due to the loss of cyclins expression and cdks activity.^28^ The regulation of cell proliferation, cell cycle and differentiation is considered an effective strategy for leukaemia treatment. Having uncovered the strong correlation between ZEB1 levels and AML disease progression, we conducted a series of cellular functional experiments to explore the underlying mechanisms. ZEB1 knock‐down and overexpression were successfully established in NB4 and THP‐1 cells. Loss‐of‐function assays demonstrated that down‐regulation of ZEB1 remarkably inhibited cell proliferation, caused cell cycle arrest and induced cell differentiation. In contrast, gain‐of‐function assays showed completely opposite findings. From this, we could conclude that ZEB1 played an important role in the pathogenesis of AML. Notably, whether ZEB1 is involved in apoptosis and drug resistance in AML cells remain to be further investigated.

Previous reports have indicated that several zinc finger transcription factors, including Snail1, Slug and Twist, directly or indirectly regulate P53 function in vitro and in vivo.^29^, ^30^ A recent literature demonstrated that ZEB1 might act as a negative upstream regulator of P53 in stromal fibroblasts and promoted mammary epithelial tumours.^20^ P53 has been the focus of human cancer research since it was first discovered from extracts of SV40‐infected animal serum by David Lane and Arnold Levine.^31^, ^32^ It took several years for people to gain a new understanding of the P53 gene. Previous reports had demonstrated that p53 is not actually an oncogene, and on the contrary, it is a major tumour suppressor gene. David lane and Arnold Levine identified p53 in the extract of SV40 transformed mouse cell line, which has become an important target in cancer treatment.^31^, ^32^ When the cells are stimulated by exogenous agents, the transcription factor p53 may show antitumour activity ^33^ but sometimes it also shows presents antagonistic phenotypes.^34^ P53 can regulate cycle arrest, senescence, apoptosis and other biological processes.^35^, ^36^ These are the most common biological functions of p53. E3 ubiquitin protein ligase MDM2 strictly controls the transcription activity of p53, which can regulate the degradation and rapid renewal of p53 in resting state.^37^ In activated cells, p53 tetramer can bind to the reactive element (RE) to regulate the expression of multiple genes related to tumour suppressor activity.^35^, ^36^, ^38^ The results show that ZEB1 can be directly combined with p53. Our results also showed that ZEB1 can inhibit the proliferation and induce the differentiation of AML cells by promoting the expression of p53. It has been shown that cell growth and proliferation can be regulated by activating cyclin D1 through the classical PI3K signalling pathway.^39^ Our results showed that ZEB1 can regulate the expression of cyclin D1 to regulate the proliferation of AML. Our previous study showed that activation of PI3K was regulated by activation of E2F4.^40^, ^41^ The above results indicate that ZEB1 may be the upstream regulatory gene of PI3K signalling pathway. When ZEB1 is activated, it may promote cell proliferation by regulating the mRNA expression and protein level of cyclin D1. Zinc finger transcription factor ZEB1 is widely recognized as an important driver of tumour invasion, distant metastasis, drug resistance and radiation resistance by regulating the induction of epithelial‐mesenchymal transition (EMT) in tumour epithelial cells.^9^, ^14^, ^42^, ^43^, ^44^, ^45^ Our next research can involve drug resistance, invasion, etc Our results also show that ZEB1 silencing can promote the expression of p53, and ZEB1 and p53 are negatively correlated. Therefore, in the treatment of AML, in addition to the above P53 activators, specific inhibitors can be developed for ZEB1. In addition, the activator of P53 is mature, but there will be side‐effects such as drug resistance. The treatment of AML is mainly divided into induction treatment stage and maintenance treatment stage. Considering the side‐effects of p53 activator, it is worth to explore whether ZEB1 inhibitor can improve the survival rate of patients in the maintenance stage and also can improve the drug resistance problems encountered in the treatment of AML. Our results also indicate that P53 silencing significantly restores the inhibitory effect of ZEB1 silencing on PI3K/AKT signalling in AML cell lines.

**FIGURE 6:**
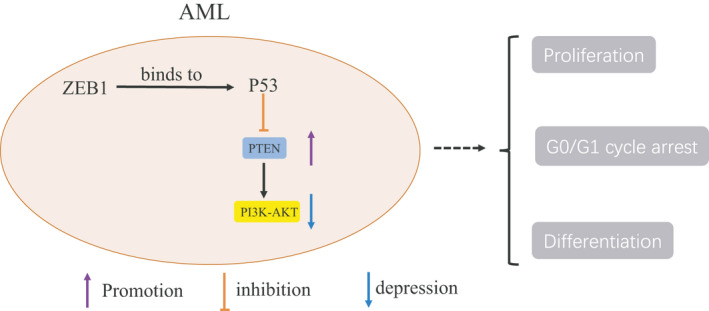
A hypothetical working model of the role of ZEB1 in AML proliferation and differentiation

As we all known, P53 is a classical tumour suppressor protein. Overall, ZEB1 can bind to P53 and regulate its expression, which may be another mechanism of ZEB1 regulating AML. The silencing of p53 significantly restored the inhibition of PI3K/AKT signalling induced by ZEB1 silencing in AML cell lines (Figure 6). In general, ZEB1 may be a new therapeutic target for AML.

## CONFLICT OF INTEREST

The authors have no conflicts of interest to disclose.

## AUTHOR CONTRIBUTIONS

**Lanlan Li:** Formal analysis (equal). **Yubin Feng:** Data curation (equal). **Shuang Hu:** Validation (equal). **Yan Du:** Conceptualization (supporting). **Xiaoling Xu:** Supervision (equal). **Meiju Zhang:** Writing‐original draft (equal). **Xiaoqing Peng:** Methodology (equal). **Feihu Chen:** Conceptualization (lead).

## Data Availability

The data used to support the findings of this study are available from the corresponding author upon request.
